# Characterization of large extracellular vesicles (L-EV) derived from human regulatory macrophages (Mreg): novel mediators in wound healing and angiogenesis?

**DOI:** 10.1186/s12967-023-03900-6

**Published:** 2023-01-30

**Authors:** Martin Albrecht, Lars Hummitzsch, Rene Rusch, Katharina Heß, Markus Steinfath, Jochen Cremer, Frank Lichte, Fred Fändrich, Rouven Berndt, Karina Zitta

**Affiliations:** 1 Department of Anesthesiology and Intensive Care Medicine, University Hospital of Schleswig-Holstein, Schwanenweg 21, 24105 Kiel, Germany; 2 Department of Anatomy, University of Kiel, Kiel, Germany; 3 Clinic of Cardiovascular Surgery, University Hospital of Schleswig-Holstein, Kiel, Germany; 4 Department of Pathology, University Hospital of Schleswig-Holstein, Kiel, Germany; 5 Clinic for Applied Cell Therapy, University Hospital of Schleswig-Holstein, Kiel, Germany

**Keywords:** Large extracellular vesicles, Macrophages, Angiogenesis, Wound healing

## Abstract

**Background:**

Large extracellular vesicles (L-EV) with a diameter between 1 and 10 µm are released by various cell types. L-EV contain and transport active molecules which are crucially involved in cell to cell communication. We have shown that secretory products of human regulatory macrophages (Mreg) bear pro-angiogenic potential in-vitro and our recent findings show that Mreg cultures also contain numerous large vesicular structures similar to L-EV with so far unknown characteristics and function.

**Aim of this study:**

To characterize the nature of Mreg-derived L-EV (L-EV_Mreg_) and to gain insights into their role in wound healing and angiogenesis.

**Methods:**

Mreg were differentiated using blood monocytes from healthy donors (N = 9) and L-EV_Mreg_ were isolated from culture supernatants by differential centrifugation. Characterization of L-EV_Mreg_ was performed by cell/vesicle analysis, brightfield/transmission electron microscopy (TEM), flow cytometry and proteome profiling arrays. The impact of L-EV_Mreg_ on wound healing and angiogenesis was evaluated by means of scratch and in-vitro tube formation assays.

**Results:**

Mreg and L-EV_Mreg_ show an average diameter of 13.73 ± 1.33 µm (volume: 1.45 ± 0.44 pl) and 7.47 ± 0.75 µm (volume: 0.22 ± 0.06 pl) respectively. Flow cytometry analyses revealed similarities between Mreg and L-EV_Mreg_ regarding their surface marker composition. However, compared to Mreg fewer L-EV_Mreg_ were positive for CD31 (P < 0.01), CD206 (P < 0.05), CD103 (P < 0.01) and CD45 (P < 0.05). Proteome profiling suggested that L-EV_Mreg_ contain abundant amounts of pro-angiogenic proteins (i.e. interleukin-8, platelet factor 4 and serpin E1). From a functional point of view L-EV_Mreg_ positively influenced in-vitro wound healing (P < 0.05) and several pro-angiogenic parameters in tube formation assays (all segment associated parameters, P < 0.05; number of meshes, P < 0.05).

**Conclusion:**

L-EV_Mreg_ with regenerative and pro-angiogenic potential can be reproducibly isolated from in-vitro cultured human regulatory macrophages. We propose that L-EV_Mreg_ could represent a putative therapeutic option for the treatment of chronic wounds and ischemia-associated diseases.

**Supplementary Information:**

The online version contains supplementary material available at 10.1186/s12967-023-03900-6.

## Introduction

Extracellular vesicles (EV), including exosomes and microvesicles, represent small, nano-to-micrometer sized structures that are released from almost all cell types of multicellular organisms [1, 2]. Growing evidence indicates that EV contain lipids, proteins and RNAs and represent an efficient way to transfer functional cargoes from cell to cell [3, 4]. Recent literature also supports a critical role for EV in mediating complex and coordinated communication among different cell types [5–7].

Apart from exosomes and microvesicles another type of EV that gained attention in the last years are so called large extracellular vesicles (L-EV). L-EV display a diameter between 1 and 10 µm and are derived and released from the cellular plasma membrane of various cell types. L-EV represent a source of bioactive molecules and due to their possible mediation of cell communication and angiogenesis they were lately also considered as potential therapeutic targets [8–13].

In this context, a special role is ascribed to the intercellular EV mediated communication between tumor cells and immune cells as tumor cells may via EV induce immune cell dysfunction to facilitate tumor proliferation and metastasis [14–16]. In addition, EV-mediated communication between endothelial cells and monocytes has been described and might play a major role in ischemia associated cardiovascular diseases: EV released from inflamed endothelial cells contain inflammatory markers, chemokines, and cytokines which are able to establish a targeted cross-talk between endothelial cells and monocytes, reprogramming the latter toward a pro- or anti-inflammatory phenotype [17]. However, EV as well as L-EV are not exclusively produced by endothelial cells but can also be secreted by other cell types such as immune cells (e.g. mast cells and macrophages) themselves [18]. Although, only few studies have focused on immune cell derived L-EV, they suggest that they may be involved in inflammatory processes and bacterial elimination [19].

As part of our search for a cell therapy-based treatment against cardiovascular ischemia-associated diseases it was shown that human monocyte derived macrophages (regulatory macrophages, Mreg) contain and release pro-angiogenic proteins and that these cells bear pro-angiogenic potential in-vitro [20]. Our recent findings also show that Mreg cultures contain numerous of vesicular structures in the size range of L-EV with unknown function.

Based on our observations and the diverse and important functions of the EV and L-EV described by other authors in the recent years we aimed at characterizing the nature of Mreg derived L-EV (L-EV_Mreg_). Furthermore, we evaluated whether the EV mediated transfer of information from endothelial cells to immune cells [17, 21, 22] also occurs in the reverse direction and whether L-EV_Mreg_ are therefore able to influence wound healing and angiogenesis in-vitro [23].

## Methods

### Mreg differentiation and isolation of large extracellular vesicles

**Fig. 1:**
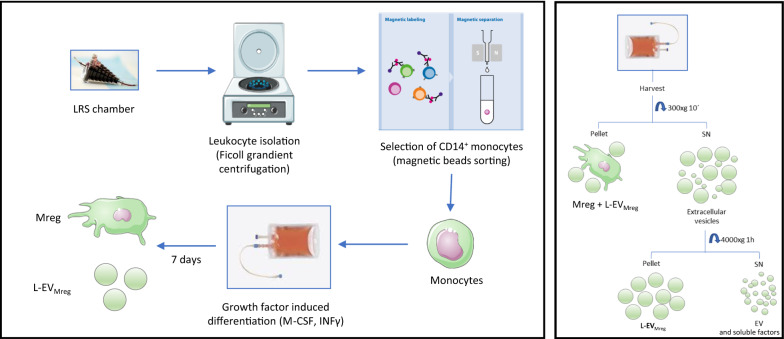
Schematic representation of in-vitro differentiation of human monocytes to Mreg and L-EV_Mreg_ (left) and differential centrifugation steps leading to L-EV_Mreg_ enriched pellets (right). SN, supernatant; EV, extracellular vesicles

The study was approved by the local Ethics Committee of the University Medical Center Schleswig–Holstein, Kiel, Germany (protocol identification: D519/18 and D518/13). Peripheral blood mononuclear cells (PBMC) were obtained from leukocyte reduction system (LRS) chambers provided by the Department of Transfusion Medicine (University Hospital of Schleswig–Holstein, Kiel, Germany) and monocytes were isolated and differentiated to Mreg (Schematic overview, Fig. 1, left). Briefly, PBMC were purified through a Ficoll-Paque PLUS gradient (GE Healthcare, Chicago, USA) and monocytes were recovered using a CD14 positive magnetic bead cell sorting system (Miltenyi, Bergisch Gladbach, Germany) following the manufacturer’s protocol. The isolated CD14 + monocytes were cultivated in bags (Miltenyi) at 0.83 × 10^6^ cells/ml with RPMI 1640 medium containing GlutaMax (GIBCO, Billings, MT, USA) supplemented with 10% human AB-Serum (Access Biological, Vista, CA, USA) and 4200 IU/ml human M-CSF (R&D Systems, Wiesbaden, Germany). The culture bags were placed in an incubator under standard culture conditions (humidified atmosphere with 5% carbon dioxide/95% air, at 37 °C). After 6 days in culture, 500 IU/ml of human interferon (IFN) γ (R&D Systems, McKinley Place MN, USA) was added to the cultures and cells were incubated for additional 24 h. On day 0 (after sell seeding) and day 6 (after addition of IFNγ) cell cultivation bags were inverted (“flipped”). On day 7 Mreg were harvested and separated from culture medium by centrifugation (300 × g for 10 min at room temperature; Fig. 1 right).

The pellets containing Mreg were resuspended in PBS and subjected to further analyses. The remaining culture supernatant containing EV was further centrifugated at 4000 × g for 1 h at 4 °C and the L-EV_Mreg_ containing pellet was resuspended in PBS and subjected to further analyses. Alternatively, L-EV_Mreg_ were resuspended 1:1 in Cryostore 5 cryopreservation medium (Stemcell technologies, Cologne, Germany) and stored at − 80 °C until further use.

### Transmission electron microscopy

Mreg and L-EV_Mreg_ pellets were fixed in 3% glutaraldehyde in PBS for 30 min. After postfixation in 2% osmiumoxide for 2 × 15 min and dehydration in increasing series of ethanol, Mreg and L-EV_Mreg_ were embedded in araldite overnight. The araldite block was trimmed for ultra-thin sectioning and ultra-thin sections. (40–50 nm) were cut using the Ultramicrotome Leica UC7 with a diamond knife (Diatom, Hatfield, PA, USA). Sections were contrasted with uranyl acetate for 15 min as well as lead citrate for 7 min. Analysis was carried out with a transmission electron microscope (Jeol JEM1400plus) coupled to a digital imaging system (TVIPS TemCam-F416).

### Automated cell and vesicle analysis

Basic parameters such as the number of particles (cells or vesicles) were evaluated using: (i) A MOXI cell counter (Orflo, Ketchum, ID, USA) which analyses membrane surrounded vesicles and cells within a size between 3 and 20 µm based on the Coulter principle. (ii) A Nucleocounter (NC-200 Chemometec, Allerod, Denmark) which stains the cell’s nucleus using 2 different dyes enabling the discrimination between live and dead cells.

### Flow cytometry

Flow cytometry was performed using the MACS Q10™ cytometer (Miltenyi). Specific antibodies and their corresponding isotypes (all from BD Biosciences) were directly conjugated with fluorescein isothiocyanate (FITC): CD31, CD16, CD45, anti-mouse IgG1κ. Conjugated with phycoerythrin (PE): CD86, CD38, CD11c, anti-mouse IgG1κ. Conjugated with allophycocyanin (APC): CD206, CD103, anti-mouse IgG1κ, CD14, anti-mouse IgG2a. The gating strategy consisted of (i) identification of the main Mreg and L-EV populations based on their size and granularity (FSC/SSC profiles), (ii) exclusion of non-viable cells (by 7-AAD exclusion, BD Biosciences), (iii) identification of Mreg using the respective identity markers and (iv) analysis of the same markers on L-EV.

### Human angiogenesis proteome profiling array

L-EV_Mreg_ were semi-quantitatively evaluated for the presence of 55 angiogenesis related proteins using a human proteome profiler array kit (R&D Systems, Minneapolis, MN, USA) as described in the manufacturer´s protocol. Briefly, isolated L-EV_Mreg_ samples resuspended in PBS were sonicated for 20 min on ice to release intravesicular proteins and were applied to the array membranes carrying antibodies against the respective pro-angiogenic proteins. After incubation, a cocktail of biotinylated antibodies and HRP-streptavidin was added to the membranes and the signals were visualized by chemiluminescence detection, referring to the manual provided. Photographs of the membranes were taken using the Fusion FX Vilber (Vilber Lourmat, Eberhardzell, Germany) and signal intensities were analyzed employing the ImageJ 1.41 software (NIH), and Vilber Smart imaging (Vilber Lourmat).

### In-vitro wound healing (“scratch assays”) and in-vitro angiogenesis (“tube formation assays”)

Human umbilical vein endothelial cells (HUVEC) were isolated from umbilical cords as described previously [24]. The cells were cultured in endothelial cell growth medium ECGM (PromoCell, Heidelberg, Germany) supplemented with 4 μL/mL of endothelial cell growth supplement, 0.1 ng/mL epidermal growth factor, 1 ng/mL basic fibroblast growth factor, 90 μg/mL heparin, 1 μg/mL hydrocortisone (all from PromoCell) and 10% fetal bovine serum (Thermo Fisher, Dreieich, Germany). HUVEC were maintained in a humidified atmosphere (5% carbon dioxide/95% air) at 37 °C. For in-vitro wound healing assays, 15.000 HUVEC /cm^2^ were seeded and grown until confluency. The cell monolayers were scratched to generate an in-vitro wound and maintained further in culture in the presence or absence of 1 × 10^6^ L-EV_Mreg_/ml. After 8 h (T8h), the size of the remaining cell-free gap was evaluated and compared to the size of the gap at T0h employing the ImageJ 1.41 software (NIH). For in-vitro tube formation assays, HUVEC were seeded in special cell culture dishes (Ibidi GmbH, Munich, Germany) according to the protocol provided by the manufacturer. Briefly, 10.000 HUVEC cells were seeded on Matrigel™ precoated wells with culture medium. After 1 h, the cells were stimulated with or without 1 × 10^6^ L-EV_Mreg_/ml. Photomicrographs of HUVEC were taken after 8 h of culture, and tube formation as well as angiogenesis related parameters were analyzed using the angiogenesis analyzer tool of the ImageJ software 1.41 (NIH) [25].

### Statistics

All experiments were carried out with L-EV_Mreg_ derived from 3 to 9 Mreg preparations from different donors. The statistics software GraphPad Prism 5.01 for windows (GraphPad Software: San Diego, USA) was used to compare groups. All data were tested for normality using the Kolmogorov–Smirnov test. In cases normality was not obtained, the data were transformed (arcsin of square root of x) and analyzed using one-way ANOVA with Tukey-test. A P-value < 0.05 was considered significant. All values are expressed as mean ± standard deviation (SD) or ± standard error of the mean (SEM) as specified in each case.

## Results

### Basic characteristics of L-EV_Mreg_

L-EV can be detected in Mreg cultures at the end of the differentiation period (day 7). The average yield of L-EV was 1.97 ± 0.64 L-EV per Mreg cell (L-EV_Mreg_/Mreg). L-EV_Mreg_/Mreg was negatively correlated with Mreg recovery (ratio of number of harvested Mreg at day 7 and number of CD14 + monocytes seeded at day 0) and lactate concentration of the culture medium, positively correlated with the pH of the culture medium and not correlated with the glucose concentration of the culture medium at day 7 (Additional file 1: Fig. S1).

**Fig. 2:**
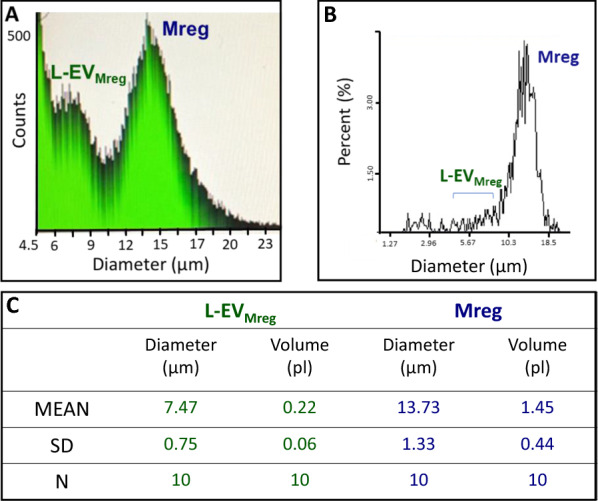
Vesicle and cell analysis. **A**. Representative analysis of a sample containing Mreg and L-EV_Mreg_ using the MOXI counter. **B**. Representative analysis of a sample containing Mreg and L-EV_Mreg_ using the Nucleocounter. Note that nucleus lacking vesicles are not detected using the Nucleocounter method. **C**. Quantitative results from vesicle and cell analysis

While Mreg reveal an average size of 13.73 ± 1.33 µm and an average volume of 1.45 ± 0.44 pl the corresponding L-EV_Mreg_ population represent definable vesicles with an average size of 7.47 ± 0.75 µm and an average volume of 0.22 ± 0.06 pl (Fig. 2). Note that while L-EV_Mreg_ are clearly detectable by methods using the Coulter counter principle (MOXI analysis; Fig. 2A), they cannot be visualized by nucleic acid staining based analysis (Nucleocounter analysis; Fig. 2B), suggesting that Mreg derived L-EV lack double stranded nucleic acids (i.e. nucleus).

### Morphology of L-EV_Mreg_

**Fig. 3:**
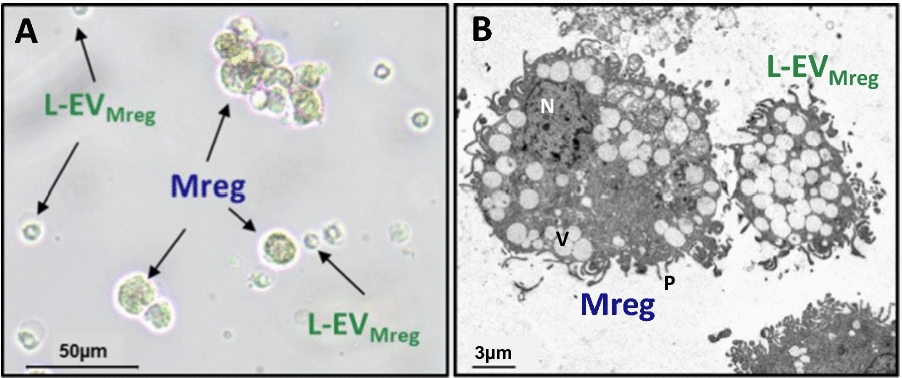
Morphology of Mreg derived L-EV (L-EV_Mreg_). **A**. Brighfield microscopy after harvest on day 7. **B**. Transmission electron microscopy after harvest on day 7. Note that L-EV_Mreg_ share several morphological features of Mreg cells such as numerous of vesicles/vacuoles and pseudopodia-like extensions. *N* nucleus, *V* vesicle/vacuole, *P* pseudopodia

L-EV_Mreg_ can easily be observed by brightfield microscopy in Mreg cultures directly after cell harvest (Fig. 3A). Employing high magnification TEM Mreg show the typical appearance of nucleated, pseudopodia containing macrophages with numerous of intracellular vesicles/vacuoles. L-EV_Mreg_ are smaller in size, lack nuclei and reveal a defined and homogeneous intravesicular structure consisting of numerous of vesicles/vacuoles in the size range around 1 µm (Fig. 3B). It should be noted that the cell culture medium containing 10% FCS does not contain any L-EV (data not shown) and that these are generated de novo during Mreg differentiation.

### Cell surface characteristics of L-EV_Mreg_

**Fig. 4:**
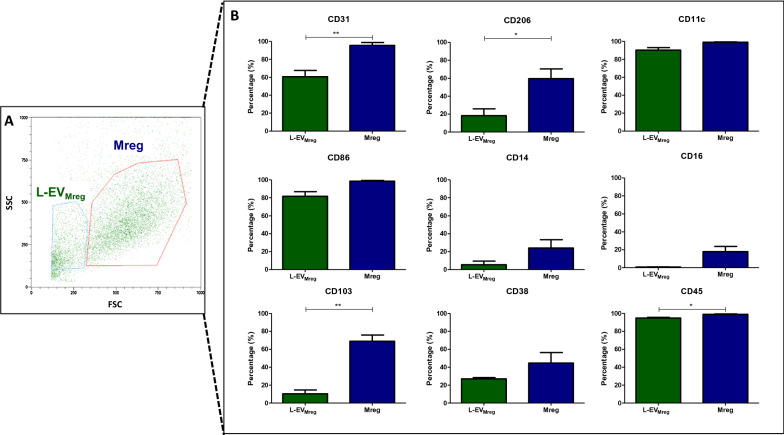
Cell surface marker characterization of Mreg derived L-EV (L-EV_Mreg_). **A**. Representative scatter plot (FCS vs. SSC) showing L-EV_Mreg_ and Mreg populations at day 7. **B**. Flow cytometric analysis of surface marker characteristics of L-EV_Mreg_ and Mreg. FSC, forward scatter; SSC, sideward scatter; *, P < 0.05; **, P < 0.01

Flow cytometry experiments were performed using 9 different antibodies against specific cluster of differentiation (CD) molecules, that were also used previously for characterization of Mreg [20]. The presence of these specific markers was analyzed on L-EV_Mreg_ as well as on Mreg, and both populations showed a similar CD expression pattern for several of the investigated molecules on their surface (CD11c on L-EV_Mreg_: 90.33 ± 2.74% and on Mreg: 99.14 ± 0.29%; CD86 on L-EV_Mreg_: 81.74 ± 5.11% and on Mreg: 98.54 ± 0.80%; CD14 on L-EV_Mreg_: 5.34 ± 4.13% and on Mreg: 24.06 ± 9.22%; CD16 on L-EV_Mreg_: 0.70 ± 0.20% and on Mreg: 17.88 ± 5.88%, and CD38 on L-EV_Mreg_: 26.93 ± 1.57% and on Mreg 44.56 ± 11.80%). However, compared to Mreg, significantly fewer L-EV_Mreg_ were positive for CD31 (L-EV_Mreg_: 60.62 ± 7.01%; Mreg: 95.50 ± 3.29%; P < 0.01), CD206 (L-EV_Mreg_: 18.26 ± 7.69%; Mreg: 59.66 ± 10.78%; P < 0.05), CD103 (L-EV_Mreg_: 10.35 ± 4.27%; Mreg: 68.92 ± 6.92%; P < 0.01) and CD45 (L-EV_Mreg_: 94.84 ± 0.86%; Mreg: 99.02 ± 0.46%; P < 0.05) (Fig. 4).

Flow cytometry analyses demonstrated the presence of typical extracellular vesicular membrane markers on the L-EV_Mreg_ (LAMP-1, CD9, CD63 and CD81) based on the guidelines for the Minimal Information for Studies of Extracellular Vesicles (MISEV 2018) [13, 26, 27], Additional file 2: Fig. S2).

### Characterization of pro-angiogenic molecules in L-EV_Mreg_

**Fig. 5:**
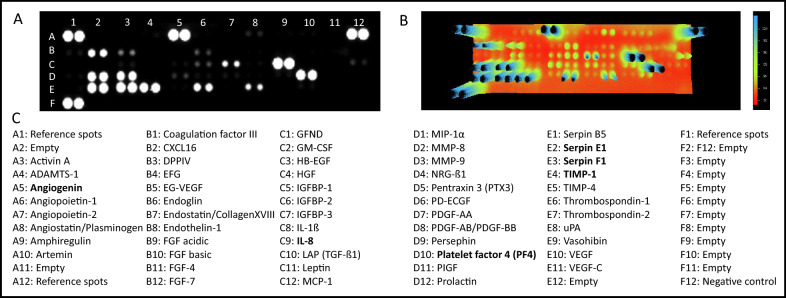
Characterization of angiogenic proteins in Mreg derived L-EV (L-EV_Mreg_). **A**. Representative proteome profiling array membrane incubated with sonicated L-EV_Mreg_ showing the relative abundance of 55 proteins involved in angiogenesis. Note that each protein is represented by duplicate spots on the respective membrane. **B**. Three-dimensional heatmap analysis of A. **C**. Arrangement of protein spots on the membrane. Proteins with the highest signal intensities are depicted in bold

Proteome profiling was performed to evaluate whether L-EV_Mreg_ contain intravesicular pro-angiogenic proteins. Semiquantitative analysis revealed the existence of abundant amounts of potentially pro-angiogenic mediators within L-EV_Mreg_. The evaluation of 55 angiogenesis related proteins showed detectable levels of 23 proteins (signal intensity > 10% of reference spots). The most abundant proteins detected in L-EV_Mreg_ were interleukin-8 (IL-8; 104.9% signal intensity compared to reference spots), platelet factor 4 (PF4; 98.5%), serpin E1 (97.2%), serpin F1 (96.5%), tissue inhibitor of metalloproteinase 1 (TIMP-1; 96.2%), and angiogenin (95.8%), Fig. 5.

### Effects of L-EV_Mreg_ on in-vitro wound healing (“scratch assays”) and in-vitro angiogenesis (“tube formation assays”)

**Fig. 6:**
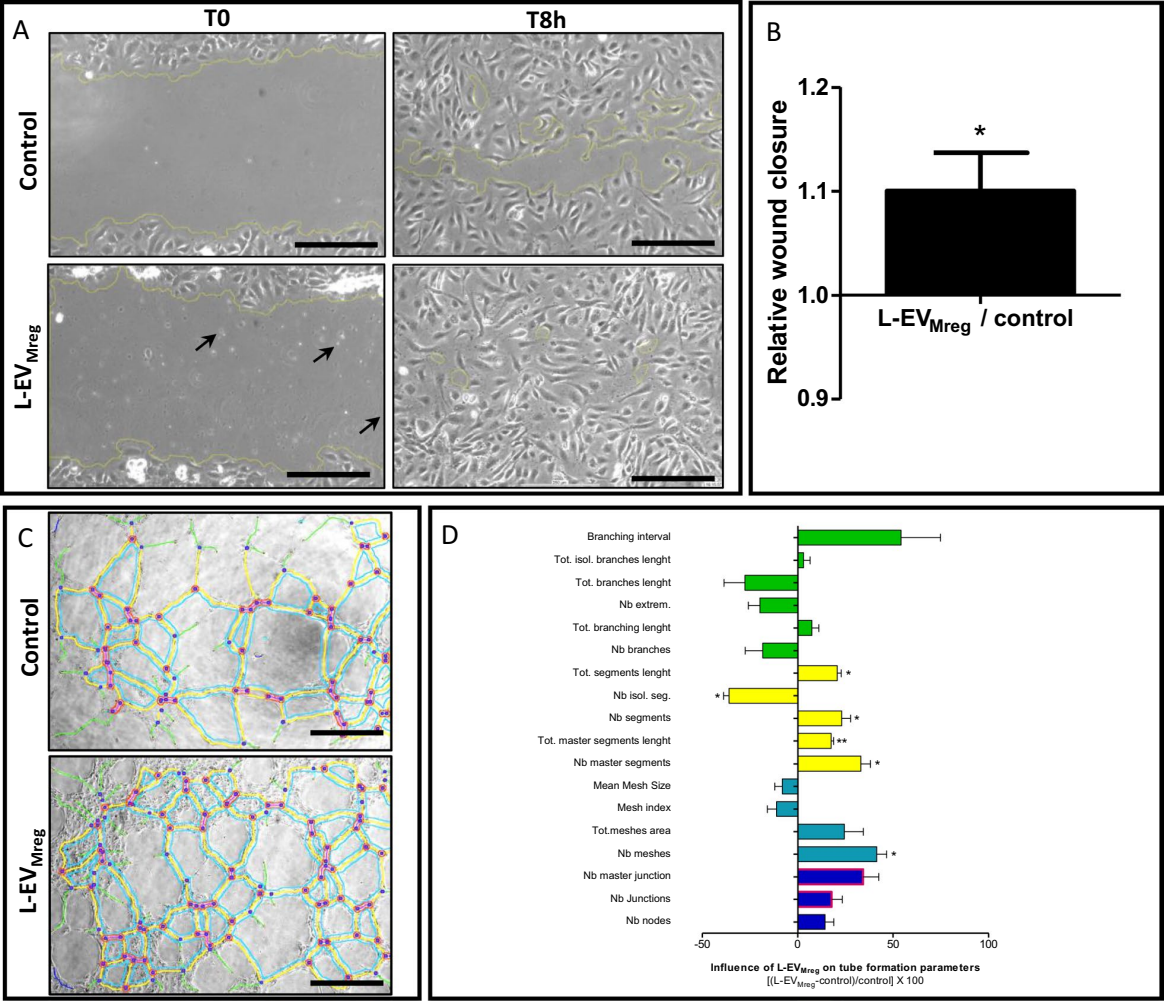
Effects of Mreg derived L-EV (L-EV_Mreg_) on in-vitro wound healing and angiogenesis. **A**. Photomicrographs of a representative wound healing assay employing human endothelial cells (HUVEC) with addition of 10^6^ L-EV_Mreg_/ml and without addition of L-EV_Mreg_. Note the presence of numerous L-EV in the treatment group (arrows). Scale bars denote 250 µm. **B**. Statistical analysis of the effect of L-EV_Mreg_ addition on relative wound closure (control = 1) after 8 h. The column denotes the mean ± SD of 3 independent experiments; *, P < 0.05 (one sample student t-test vs. 1). **C**. Representative images of tube formation analysis employing HUVEC cell cultures with and without the addition of L-EV_Mreg_. Scale bars denote 500 µm. **D**. The influence of L-EV_Mreg_ addition on various tube formation parameters. Horizontal columns denote the mean ± SD of 3 independent experiments. *, P < 0.05; **, P < 0.01 (one sample student t-test vs. 0). Green color, branches related parameters; yellow color, segments related parameters; cyan color, meshes related parameters; blue color, nodes related parameters; red color, junctions related parameters. For parameter definitions and details on the angiogenesis analyzer tool please refer to: http://image.bio.methods.free.fr/ImageJ/?Angiogenesis-Analyzer-for-ImageJ&lang=en

To investigate weather L-EV_Mreg_ are able to positively influence wound healing and angiogenesis, scratch as well as tube formation assays using human endothelial cells (HUVEC) were performed. Scratch assays revealed that the presence of L-EV_Mreg_ resulted in a slightly higher percentage of endothelial cell covered area compared to control, indicating positive effects of L-EV_Mreg_ on wound healing (L-EV_Mreg_: 86.64 ± 11.67%; control without L-EV_Mreg_: 78.65 ± 8.58%; ratio L-EV_Mreg_/control: 1.10 ± 0.04; P < 0.05; Fig. 6A, B). Tube formation assays showed that the presence of L-EV_Mreg_ during the culture period significantly influenced 6 out of the 18 investigated and angiogenesis associated parameters (total of segment length: + 20.70 ± 2.12%; P < 0.05; number of isolated segments: − 36.10 ± 2.78%; P < 0.05; number of segments: + 23.00 ± 4.68%; P < 0.05; total of master segment length: + 17.50 ± 1.30%; P < 0.01; number of master segments: + 33.10 ± 4.96%; P < 0.05; number of meshes: + 41.30 ± 5.18%; P < 0.05; Fig. 6C, D).

## Discussion

Extracellular vesicles (EV) are produced and released by almost all cell types of multicellular organisms and they have been detected in various biological fluids such as blood [28], urine [29], saliva [30] and breast milk [31]. EV can be classified by size, from small (< 1 µm) to large (1 µm–10 µm) vesicles [32] or by their biogenesis and mode of secretion (i.e. via endosomal sorting complex or direct budding from the cell membrane) [32]. The molecular mechanisms involved in EV formation and release are complex and still not completely understood [32–34]. From a functional point of view, EV are an efficient way to transfer cargoes such as lipids, metabolites, proteins and nucleic acids from cell to cell [35]. Moreover, EV are involved in coordinated communication among different cell types [5–7]. The most studied EV are exosomes and microvesicles (50–200 nm), as well as apoptotic bodies (1–5 µm) [36]. The observation that much larger EV (L-EV) of up to 10 µm exist and may play a role in tumor progression has driven the scientific focus more towards L-EV in recent years [37, 38]. An indispensable prerequisite of tumor progression is the adequate supply of nutrients, which is usually achieved by enhanced angiogenesis in the tumor area [39].The hypothesis that L-EV exert pro-angiogenic effects not only in tumor areas is very tempting and L-EV may bear therapeutic potential for the treatment of ischemia-associated diseases and wound healing processes that require an increased angiogenesis.

In the last years, our group has been working intensively on a cell therapy-based approach for the treatment of ischemia-associated cardiovascular diseases. In this context, we showed that regulatory macrophages (Mregs), which can be differentiated under in-vitro conditions from peripheral blood monocytes, have several distinct and clinically relevant properties including immunomodulatory, anti-inflammatory, and potentially angiogenic/tissue regenerative effects. The latter are possibly mediated through the upregulated secretion of pro-angiogenic factors under hypoxic conditions [20]. Interestingly, at the end of the differentiation period in-vitro, Mreg cultures contain numerous of vesicular structures, from which L-EV_Mreg_ can be isolated. As Mreg bear pro-angiogenic potential, the aim of this study was to characterize the nature of L-EV_Mreg_ and their possible function in wound healing and angiogenesis.

It is already known that macrophages are able to release vesicular structures. Macrophage-derived smaller vesicles were reported to transport and transfer various bioactive molecules emerging as vital mediators of beneficial effects in immunomodulation and tissue repair [8, 9]. Moreover, inflammatory macrophage-derived exosomes can reprogram neighboring macrophages polarizing them into an anti-inflammatory phenotype, promoting wound healing [40]. However, there are only very few studies that have described and characterized macrophage derived L-EV mainly relating them to inflammatory functions [10, 19].

Morphologically, L-EV_Mreg_ resemble well the so far described L-EV of other cell types: L-EV_Mreg_ represent definable membrane surrounded vesicles with an average size of 7.47 µm and an average volume of 0.22 pl. They possess typical surface CD markers of vesicular structures (e.g. CD63, CD9, CD81 and LAMP-1) [26] and preliminary analyses revealed that the release of L-EV_Mreg_ per Mreg cell is negatively correlated with the percentage of Mreg recovery at day 7 and lactate concentration of the culture medium, positively correlated with the pH of the culture medium and not correlated to the glucose concentration of the culture medium. These findings may be of clinical/therapeutic interest in that they suggest that the yield of L-EV_Mreg_ can to some extent be controlled by the composition of the culture medium and in particular by the pH level.

TEM and nucleic acid staining suggest that L-EV_Mreg_ are deficient of nuclei and show numerous intravesicular vesicles/vacuoles in the size range around 1 µm which are also present in Mreg. Regarding the presence of typical cell surface CD molecules that characterize potentially pro-angiogenic Mreg [20] it was shown that these markers are also detected on L-EV_Mreg_ suggesting that L-EV_Mreg_ might resemble structures that are generated by budding from the Mreg cells. Interestingly, compared to Mreg, less L-EV_Mreg_ revealed to be positive for CD206 [41], CD31 [42], and CD103 [43]. This observation is of interest as it may suggest that active processes are involved in the production/sorting of the respective L-EV_Mreg_ and that their composition and thus physiological properties may be directly controlled by Mreg cells themselves.

Based on the composition of the surface CD molecules, which was relatively similar to that of Mreg, we further investigated the contents of L-EV_Mreg_ by angiogenesis proteome profiling assays. 23 of the 55 proteins analyzed were detected in L-EV_Mreg_. Of these, 6 proteins were found in large amounts (more than 90% signal intensity compared to the reference spots): interleukin-8, platelet factor-4, serpin E1, serpin F1, TIMP-1, and angiogenin. Interestingly, these proteins are also produced and secreted de novo when Mreg are cultured in plastic dishes for 24 h after harvest [20]. With regard to angiogenesis, looking at the 6 factors individually does not provide a uniform picture: interleukin-8 and angiogenin are potent stimulators of angiogenesis [44, 45], while platelet factor-4 is mainly known as angiogenesis inhibitor [46]. Serpin E1 (PAI-1) appears to exert pro- or antiangiogenic functions [47] and the ubiquitous and multifunctional proteins serpin F1 and TIMP-1 show as well ambivalent functions regarding angiogenesis [48–50]. However, it can be assumed that not a single factor but rather the combination of intravesicular factors together with the membrane-bound CD molecules and receptor composition on or within the target cells (i.e. endothelial cells) is responsible for the observed pro-angiogenic in-vitro results and possible pro-angiogenic effects of L-EV_Mreg_ in-vivo.

A potential influence of L-EV_Mreg_ on wound healing and angiogenesis was further analyzed by scratch assays and tube formation assays using human endothelial cells. Both assays have already been successfully used by numerous other groups and reliably provide insights into possible regenerative or pro-angiogenic effects of the investigated compounds in-vitro [51]. At a concentration of 1 million L-EV_Mreg_ per ml, significantly positive effects were observed on both in-vitro wound healing and in-vitro angiogenesis. Interestingly, our previous studies with monocytes and Mreg showed that neither monocyte cell culture supernatants nor Mreg culture supernatants were able to exert significant effects on in-vitro tube formation representative of angiogenesis [20, 52]. In contrast, the results presented here suggest that the L-EV_Mreg_ fraction, unlike the total cell culture supernatants, may be able to positively influence wound healing and angiogenesis. The extent to which these encouraging findings are transferable to the in-vivo situation remains however elusive and requires further studies.

There are some limitations of the study that need to be addressed. Based on the results of a smaller series of pilot experiments the most effective and promising concentration of L-EV_Mreg_ (1 million/ml) was used in the in-vitro wound healing and angiogenesis experiments. Whether this concentration can also be applied to the in-vivo situation is yet unclear and has to be evaluated in future animal experiments and clinical studies. As the differentiation process of Mreg requires supplementation with 10% human AB serum, compliance with MISEV guidelines [26] regarding the use of serum for the preparation of L-EV_Mreg_ was not possible. Nevertheless, it could be shown that the serum used was free of L-EV and that the L-EV_Mreg_ described in the present work arose de novo during the 7-day differentiation period. Finally, although we are aware of the pure in-vitro nature of the study and all associated limitation, to the best of our knowledge, this is the first study to show that L-EV with regenerative and pro-angiogenic potential can be reproducibly produced from in-vitro cultured human regulatory macrophages.

## Conclusion

Large extracellular vesicles are generated during the in-vitro differentiation of monocytes to regulatory macrophages (Mreg) and could bear therapeutic potential for the treatment of chronic wounds and ischemia-associated diseases such as peripheral arterial occlusive disease.

## Supplementary Information

**Additional file 1** Correlation of L-EV_Mreg_/Mreg ratios with cell culture media characteristics at harvest.

**Additional file 2** Representative flow cytometry for vesicular biomarkers.

## Data Availability

The datasets used and/or analysed during the current study are available from the corresponding author on reasonable request.
