# Supplementary material for: Characterization of large extracellular vesicles (L-EV) derived from human regulatory macrophages (Mreg): novel mediators in wound healing and angiogenesis?
Source: J Transl Med. 2023 Jan 30;21:61. doi: 10.1186/s12967-023-03900-6 (PMC9887800; doi:10.1186/s12967-023-03900-6)
Supplement: Supplementary file 1 — Additional file 1 Correlation of L-EVMreg/Mreg ratios with cell culture media characteristics at harvest. [file 12967_2023_3900_MOESM1_ESM.pptx]

## Slide 1
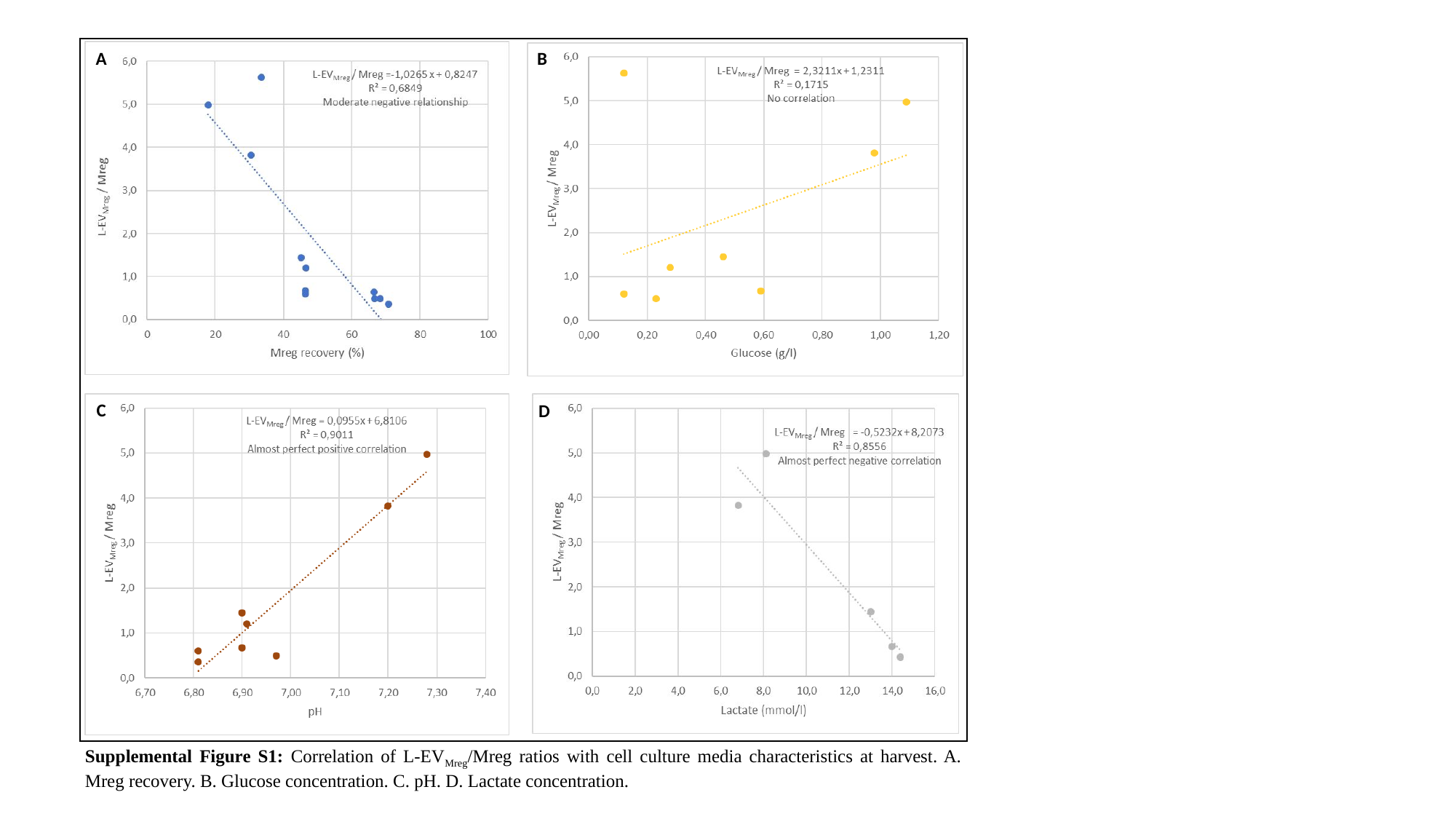

B
A
C
D
Supplemental Figure S1: Correlation of L-EVMreg/Mreg ratios with cell culture media characteristics at harvest. A. Mreg recovery. B. Glucose concentration. C. pH. D. Lactate concentration.
