# Supplementary material for: Characterization of large extracellular vesicles (L-EV) derived from human regulatory macrophages (Mreg): novel mediators in wound healing and angiogenesis?
Source: J Transl Med. 2023 Jan 30;21:61. doi: 10.1186/s12967-023-03900-6 (PMC9887800; doi:10.1186/s12967-023-03900-6)
Supplement: Supplementary file 2 — Additional file 2 Representative flow cytometry for vesicular biomarkers. [file 12967_2023_3900_MOESM2_ESM.pptx]

## Slide 1
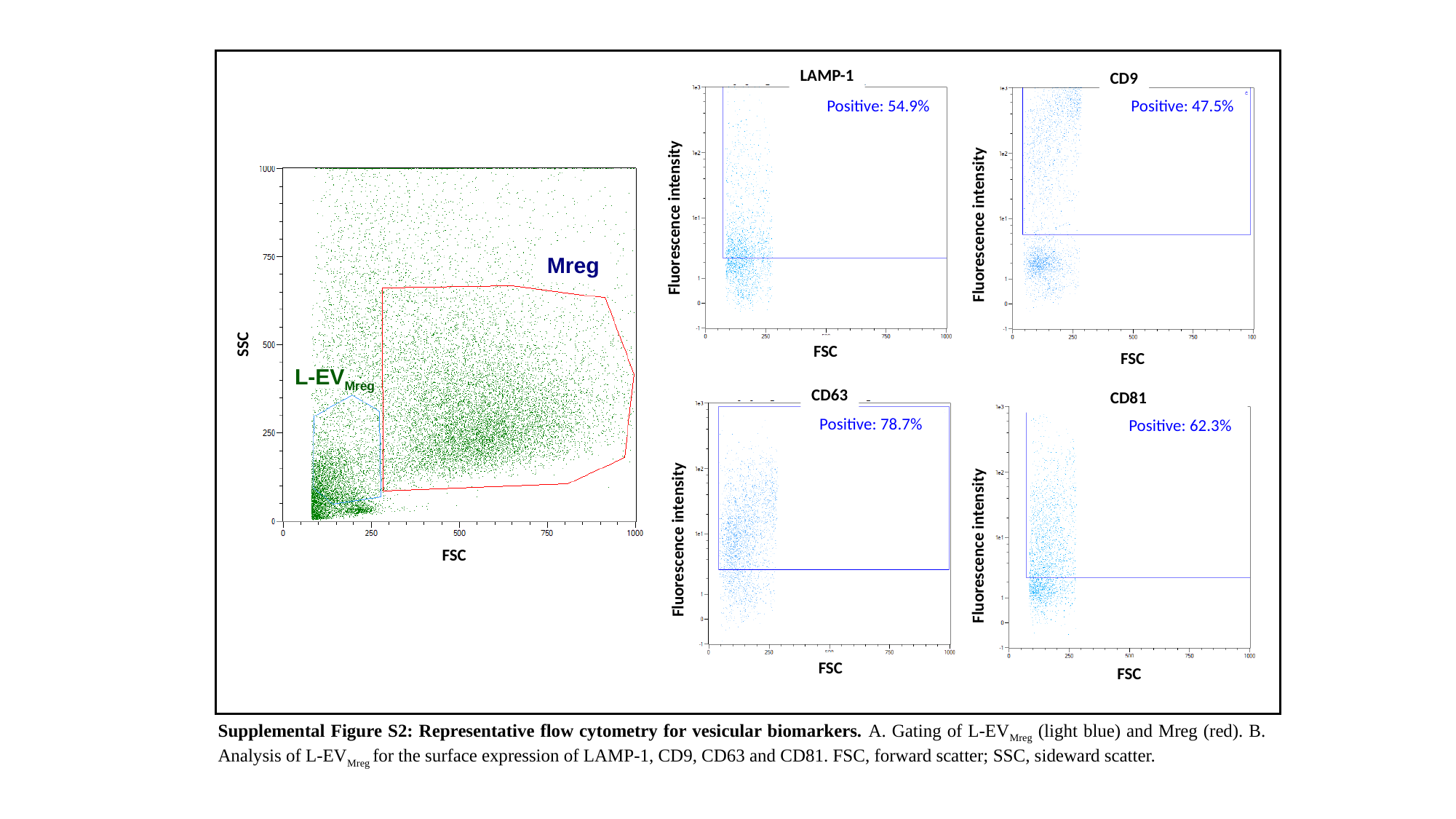

LAMP-1
Positive: 54.9%
Fluorescence intensity
FSC
CD9
Positive: 47.5%
Fluorescence intensity
FSC
SSC
FSC
Mreg
L-EVMreg
CD63
Positive: 78.7%
Fluorescence intensity
FSC
CD81
Positive: 62.3%
FSC
Fluorescence intensity
Supplemental Figure S2: Representative flow cytometry for vesicular biomarkers. A. Gating of L-EVMreg (light blue) and Mreg (red). B. Analysis of L-EVMreg for the surface expression of LAMP-1, CD9, CD63 and CD81. FSC, forward scatter; SSC, sideward scatter.
